# Preclinical evaluation of a hydraulic actuation system with guide tube for robotic cochlear implant electrode insertion

**DOI:** 10.1186/s12938-025-01338-z

**Published:** 2025-02-14

**Authors:** Jakob Cramer, Rolf Salcher, Max Fröhlich, Georg Böttcher-Rebmann, Eralp Artukarslan, Thomas Lenarz, Thomas S. Rau

**Affiliations:** 1 Department of Otolaryngology and Cluster of Excellence EXC 2177/1 “Hearing4all”, Hannover Medical School, Carl-Neuberg-Str. 1, 30625 Hannover, Germany; 2 Lower Saxony Center for Biomedical Engineering, Implant Research and Development (NIFE), Hannover, Germany; 3 MED-EL Research Center, Hannover, Germany

**Keywords:** Automated electrode insertion, Guide tube, Ex vivo insertion trials, Motion tracking system, Automated vs manual actuation, Surgical tool

## Abstract

**Background:**

Automated insertion of the cochlear implant electrode array can reduce the risk of intracochlear trauma. To address this, our group previously developed a hydraulic electrode insertion device, the Cochlea Hydrodrive (CHD), which automates the process using a syringe piston driven by an infusion pump. This study aims to characterize the hydraulic actuation process of the CHD and to preclinically evaluate its design.

**Methods:**

A camera-based motion tracking test setup was developed to obtain hydraulic motion profiles. Various syringes were evaluated for their actuation properties and the optimal syringe was selected. The CHD design was adapted based on the selected syringe, incorporating a slotted stainless steel guide tube to surround the electrode during insertion. This enhanced design was tested in ex vivo insertion trials into human head specimens.

**Results:**

The final design of the CHD demonstrated smooth and steady motion profiles at all tested velocities (0.4 mm/s, 0.1 mm/s, 0.03 mm/s). Ex vivo insertion trials confirmed these findings, with the guide tube facilitating easy alignment of the CHD in front of the round window and preventing electrode buckling.

**Conclusion:**

Our study validates that the CHD provides reliably smooth actuation properties despite its low complexity. The use of a guide tube appears promising and could further enhance the standardization of automated electrode insertion.

**Supplementary Information:**

The online version contains supplementary material available at 10.1186/s12938-025-01338-z.

## Introduction

The cochlear implant (CI) is the standard of care to restore hearing in patients with severe to profound hearing loss. While initially only deaf patients were treated with a CI, the indication has gradually been expanded to treat patients with residual hearing [1]. Despite the good hearing outcomes [2, 3], preservation of residual hearing cannot yet be guaranteed [4] and many potential CI candidates reject treatment due to the fear of losing their residual hearing [5]. This is why, over the past few decades, a major research focus in the field of CI surgery has been on making the procedure less traumatic and thereby preserving as much residual hearing as possible. It was found that the insertion process of the electrode array (EA) of the CI into the scala tympani is a critical step and a common cause for intracochlear trauma [6, 7]. Different approaches have been developed to reduce implantation trauma, including intracochlear drug application [8], monitoring of hair cell responses [9], or the analysis of the individual cochlear geometry and choice of EA, as this is known to have an impact on insertion forces [10, 11]. Clinical data and temporal bone studies further suggest, that controlling the insertion speed reduces the risk for intracochlear trauma [12–16]. This is supported by laboratory experiments indicating a significant dependence of insertion speed, forces [17–21] and pressure transients [22, 23]. Automated EA insertion devices are one approach to combine the best practices of well experienced surgeons and laboratory findings and translate them into clinical treatment. Those robotic systems ensure steady insertion under controlled velocity, offering a high degree of standardization, which makes the insertion process less dependent on experience and manual dexterity of the surgeon [15, 24]. However, current electromechanical, automated insertion devices available on the market are rather complex and cost-intensive [25, 26].

To address these challenges, our group recently developed a hydraulically driven, automated insertion tool characterized by its simplicity and therefore cost-efficient application, the *Cochlea Hydrodrive* (CHD) [27]. The system uses a common infusion pump to drive a second syringe repurposed as a hydraulic piston. An EA holder is attached to the piston, and the whole system is fixed to the patient using a surgical retractor and a flexible arm. In our previous work, we demonstrated the general feasibility of the hydraulic principle, as well as the ease of application and handling for EA insertions into human head specimens [27–29].

The scope of this work consists of three main objectives. First, to develop and evaluate a motion tracking test setup in order to characterize the hydraulic actuation process of the CHD. Second, to select the best syringe for actuation of the CHD system based on experimentally evaluated motion profiles. Lastly, to adjust the CHD design based on the selected syringe, enhance the system with a guiding mechanism to prevent buckling by surrounding the EA during the insertion process, and evaluate this updated CHD design in ex vivo EA insertion trials.

## Material and methods

### Tracking test setup

#### Test setup design and motion tracking

**Fig. 1:**
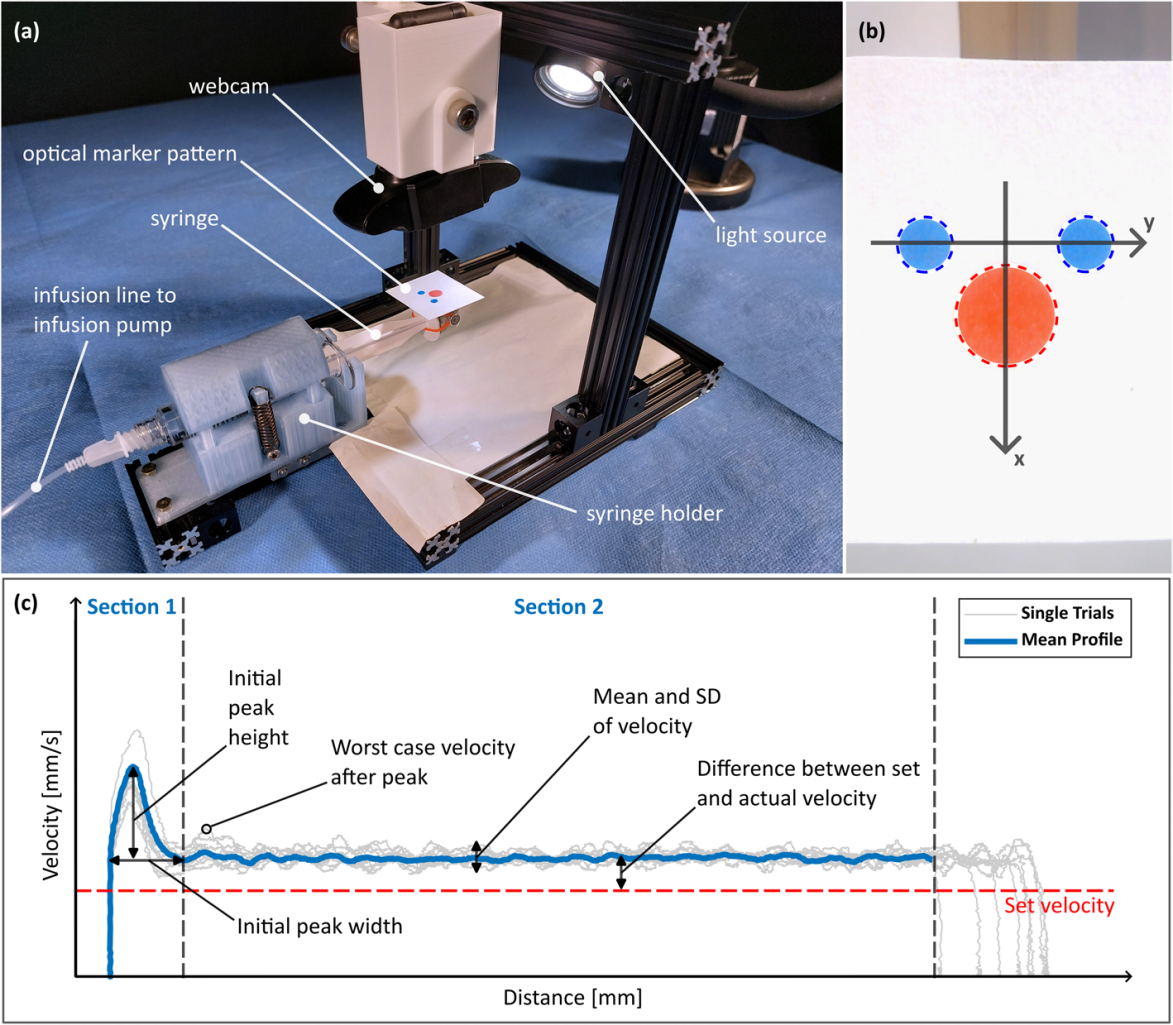
**a** Tracking test setup with its components; **b** video image including the tracked circle markers (dashed lines) and coordinate system with x-axis as direction of travel; **c** representative movement profile with evaluation parameters for syringe selection

In order to evaluate the actuator movement of the hydraulic system, a motion tracking system was developed including a universal syringe holder, a webcam (Conceptronic AMDIS02B, Digital Data Communications GmbH, Dortmund, Germany) and an optical marker pattern attached to the syringe plunger (see Fig. 1a). The setup allows video-recording (720p, 30fps) of the movement of the optical marker pattern on the plunger, initiated by the connected infusion pump (Perfusor^®^ fm, BBraun SE, Melsungen, Germany).

Post-processing of the captured videos and position tracking were executed in Matlab (R2024a, The MathWorks, Natick, USA). First, to mitigate errors in position tracking arising from camera lens distortion, the used camera was calibrated with a checkerboard image to determine its intrinsic parameters. This calibration was performed using Matlab’s *image processing and computer vision toolbox*. The resulting calibration parameters were then applied to correct the recorded images.

Using the Matlab script, videos were analyzed frame by frame. Within each frame, the circles of the marker pattern were segmented using color thresholds based on an YCbCr color filter. Subsequently, the centers of the segmented circles were determined. The optical marker pattern was used to create a conversion factor from pixels to millimeters and to define a coordinate system to identify the position and movement direction of the syringe plunger within the picture (see Fig. 1b). By utilizing the frame rate of the video and the calculated distance traveled, the displacement–time profile could be calculated from this data. Following this, the velocity–time profile was derived from the displacement–time profile using differentiation, specifically by calculating the gradient over a time window of 0.5 s (15 frames).

#### Test setup evaluation

Evaluation experiments were conducted to assess the accuracy of the above described method. For this purpose, the tracking pattern was mounted on a motorized, programmable linear stage (LTM 45–110-HiSM, OWIS GmbH, Staufen im Breisgau, Germany) with a position error of less than 35 µm per 100 mm. The set motion profile on the linear stage consisted of four distinct velocities: 1.0 mm/s, 0.4 mm/s, 0.1 mm/s and 0.03 mm/s. Each velocity was maintained for ten seconds, with a two seconds pause between sections. This process was repeated ten times. Finally, the agreement of the designated movement profile and the tracked movement profile, i.e., the accuracy of the motion tracking method, was analyzed. This was done using linear correlation and Bland–Altman analysis [30].

### Syringe selection and hydraulic actuation

#### Identification of suitable syringe models for evaluation experiments

Suitable syringes for the hydraulic system were identified from a product search. The inclusion criteria required the disposable syringes to possess a Luer-lock connection for secure attachment to an infusion line and to be approved for use on humans. Additionally, the selected models should be tripartite, consisting of a barrel and a plunger with seal ring, as we expected these models to provide better friction conditions due to the seal. Syringes with a volume exceeding 6 ml were excluded to ensure that the system remains as compact as possible. In total 11 syringe models from four different manufacturers were identified (see Table 1).

**Table 1:** Identified syringe models and sizes with corresponding abbreviations

Syringe model	Volume size	Abbreviation
^1^Omnifix^®^	5 ml	BBO5
	3 ml	BBO3
	1 ml	BBO1
^2^BD Plastipak^™^	3 ml	BDP3
	1 ml	BDP1
^3^Medallion^®^	6 ml	MMM6
	3 ml	MMM3
	1 ml	MMM1
^4^ClearJect^®^	5 ml	GCJ5
	2.25 ml	GCJ225
	1 ml	GCJ1

^1^BBraun SE, Meslungen, Germany

^2^Becton Dickinson GmbH, Heidelberg, Germany

^3^Merit Medical Inc., South Jordan, USA

^4^Gerresheimer AG, Duesseldorf, Germany

#### Evaluation parameters

An objective evaluation rating system was introduced to assess the actuation behavior of each tested syringe model, based on their specific tracked motion profile (see Fig. 1c). First, the average speed profile across all actuation trials was calculated. This profile was then partitioned into two evaluation sections. The first section describes an initial velocity peak, which may arise from overcoming static friction between barrel and plunger. The second section extends from the end of the initial peak to the end of the actuation, just before the velocity drops. For both sections, evaluation parameters and corresponding weighting factors (WF) were devised.

Within the first section, evaluation parameters contain the height (WF: 0.1) and width (WF: 0.2) of the mean initial peak. In the second section, three additional parameters were examined: the difference between the target and actual mean velocity (WF: 0.2), the maximum velocity deviation exceeding the targeted speed (worst case) of a single trial (WF: 0.25) and the highest standard deviation observed across all trials for all values within the second section (WF: 0.25). For all parameters, smaller values indicate better performance. Separate rankings were compiled for each evaluation parameter. The syringes were ranked from low to high. The ranks for each parameter were then multiplied with the respective weighting factor and summed up to obtain a cumulative rating for each syringe.

#### Experimental study design

The syringes were tested in three evaluation stages with differing velocities. In the first stage, a velocity of 0.4 mm/s was employed, followed by a velocity of 0.1 mm/s in the second stage, and a further reduction to 0.03 mm/s in the third stage. The corresponding flow rate at the infusion pump ($$V)$$ was calculated for every syringe using the general equation for the average volume flow (see Eq. 1), with $$v$$ as desired actuator velocity and $$r$$ as the measured diameter of the syringe plunger:

1 $$V = v*\pi *{r}^{2}.$$

During each stage, the actuation of every syringe model was measured six times using the motion tracking setup with the syringe being replaced after three trials. The actuation distance was set to 28 mm as this is the length of a commonly implanted long flexible lateral wall EA. The study was designed with a “survival-of-the-fittest” approach. After every stage, syringes were evaluated and ranked regarding the aforementioned parameters. The best ranked half of the syringes were transferred to the next stage while the other syringes got eliminated from the experiments. The best syringe of the last step was then selected for future application in the CHD system.

For the direct comparison, additional manual actuation trials were performed. Four different test persons were instructed to perform the forward motion as slowly and steadily as possible during five insertions of an EA (Flex28, MED-EL, Innsbruck) into a cylinder with a diameter of 5 mm using a surgical forceps. For manual velocity tracking and evaluation, the previously introduced test setup was used.

### Advancement of the Cochlea Hydrodrive design

#### Design changes to the Cochlea Hydrodrive system

Design of all components of the original CHD system [27] was slightly revised in order to reduce its size. Furthermore, the system was enhanced with an optional guiding mechanism. This feature is intended to serve two primary functions: first, to surround the EA during the insertion process to minimize uncontrolled buckling, and second, to support the alignment of the CHD at the round window along the desired insertion trajectory. Designing a version with a guiding mechanism was supported by preliminary tests on human cadaver head specimens that aimed to evaluate the general functional principle (as described below). These findings have been considered in the following development towards the final design.

#### Experimental evaluation of the advanced design

The experimental evaluation of the adapted CHD design comprised two parts. One aim was to assess whether the additional friction between the guiding mechanism and the electrode holder had any potentially negative effects on the uniformity of the motion. Using the same motion tracking method, velocities of 0.4 mm/s, 0.1 mm/s and 0.03 mm/s were tested, with 10 trials for each velocity and with the syringe being replaced after every trial.

Additional experiments were performed to evaluate the handling of the system with the guiding mechanism in a realistic environment. Here, a 3D printed prototype version of the CHD was used, which had the same functional dimensions as the final manufactured design. To achieve this handling evaluation, insertion experiments into human head specimens were performed. The cadaver head underwent preparation for CI implantation including a mastoidectomy, posterior tympanotomy, manipulation at the bony overhang, and opening of the round window membrane. Two FLEX28 electrode arrays (MED-EL, Innsbruck, Austria) composed of platinum contacts and wires, embedded in a silicone matrix [31], were used for insertion trials. The arrays were over-molded with a silicone processor dummy to resemble realistic dimensions and post-insertion lead management of the SYNCHRONY 2 implant. Prior to every insertion, the scala tympani was flushed with NaCl to ensure intracochlear lubrication in order to improve friction properties of the preserved cochlear specimens. For the insertion, the CHD was fixed to the head specimen using a surgical retractor (Anderson-Adson Retractor, Aspen Surgical, Nashville, USA) and a flexible arm (Greenberg^®^ Retractor LongArm, Aspen Surgical, Nashville, USA), as intended. Afterwards, the EA was clamped into the electrode holder of the CHD, which was subsequently aligned in a way that the tip of the EA was positioned directly in front of the round window. When using the CHD with the optional guiding mechanism, the system was loaded with the EA and the guiding mechanism was placed directly in front of the round window. After alignment, the flexible arm was stiffened using the adjusting screw. Two senior surgeons from our clinic performed three insertions in total using the CHD without and four insertions with the additional guiding mechanism with an insertion speed of 0.4 mm/s. After the insertion, the EA was released from the electrode holder using a surgical needle, transferred and fixed into a bone grove within the facial recess. Before the automated procedure, a manual reference insertion was performed to determine the maximal possible insertion depth in the cadaver specimen. Following each insertion, a CBCT scan was acquired (xCAT, Xoran Technologies LLC, Ann Arbor, Michigan) to obtain 3D imaging of the inserted EA.

## Results

### Evaluation of the tracking test setup

**Fig. 2:**
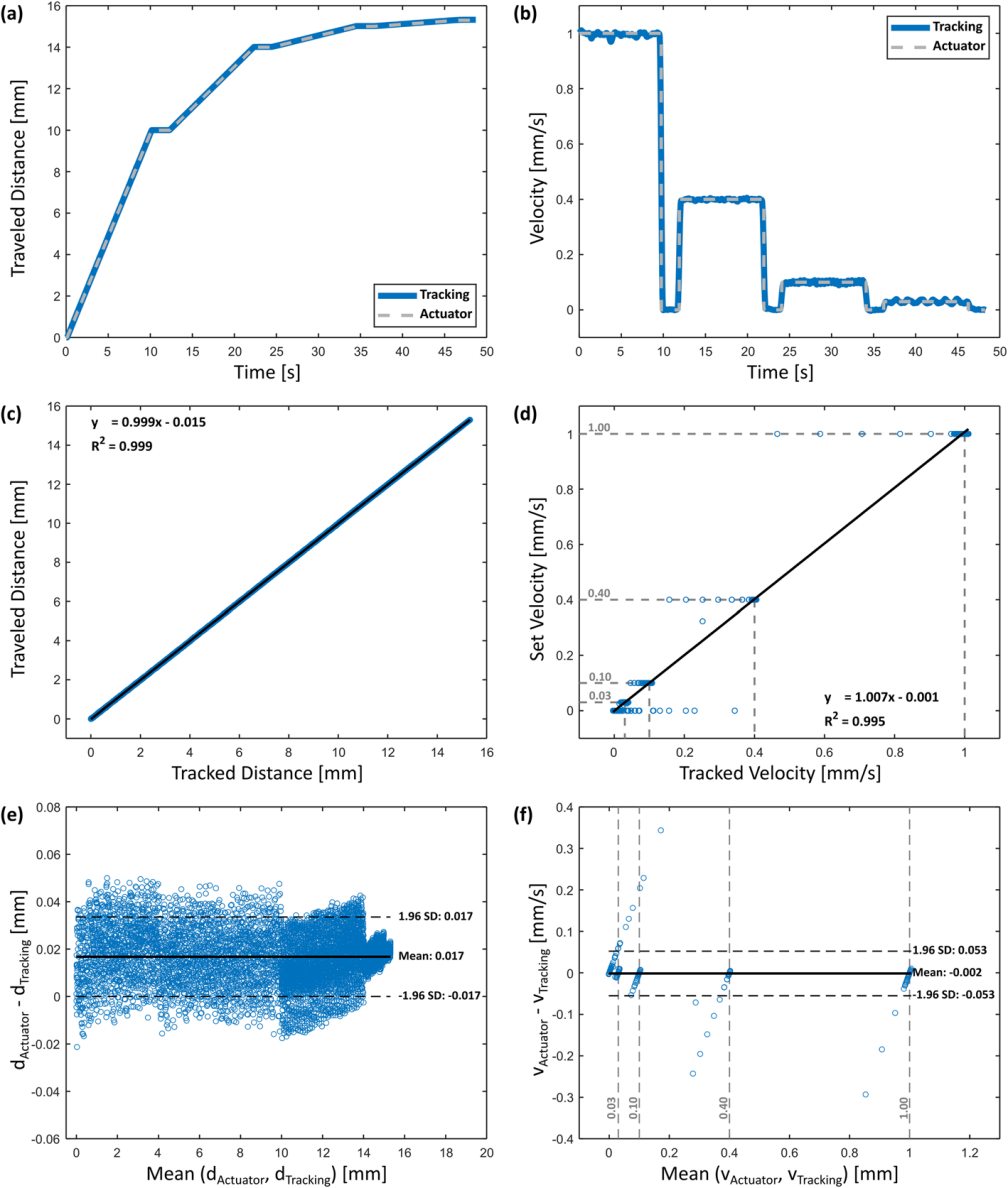
Results of the test setup evaluation. Comparison of actuator and tracked data for **a** mean distance and **b** mean velocity. Correlation plots for **c** distance and **d** velocity evaluation. Bland–Altman plots of **e** distance and **f** velocity

The results of the evaluation experiments revealed a close correspondence between the target motion profiles realized by the linear stage and those tracked using the motion tracking test setup. This alignment was evident both in the tracked distance and in the calculated speed profiles (see Fig. 2a and b) and could further be observed in the correlation plots (see Fig. 2c and d), containing measurement points from all ten trials. Here, a strong correlation is demonstrated for the distance tracking (*R*^2^ = 0.999) and for the velocity tracking (*R*^2^ = 0.995), respectively. The Bland–Altman diagrams reveal similarly precise results with a mean difference of 0.017 mm between the distance signals, with a confidence interval of ± 0.017 mm, and a mean difference of − 0.002 mm/s between the velocity signals, with a confidence interval of ± 0.053 mm/s (see Fig. 2e and f). Notably, outliers in the velocity tracking were observed before and after sudden velocity changes in both the correlation plot and the Bland–Altman plot. Nonetheless, the high correlation values indicate a strong agreement between the signals during the investigated velocities.

### Syringe selection and hydraulic actuation

**Fig. 3:**
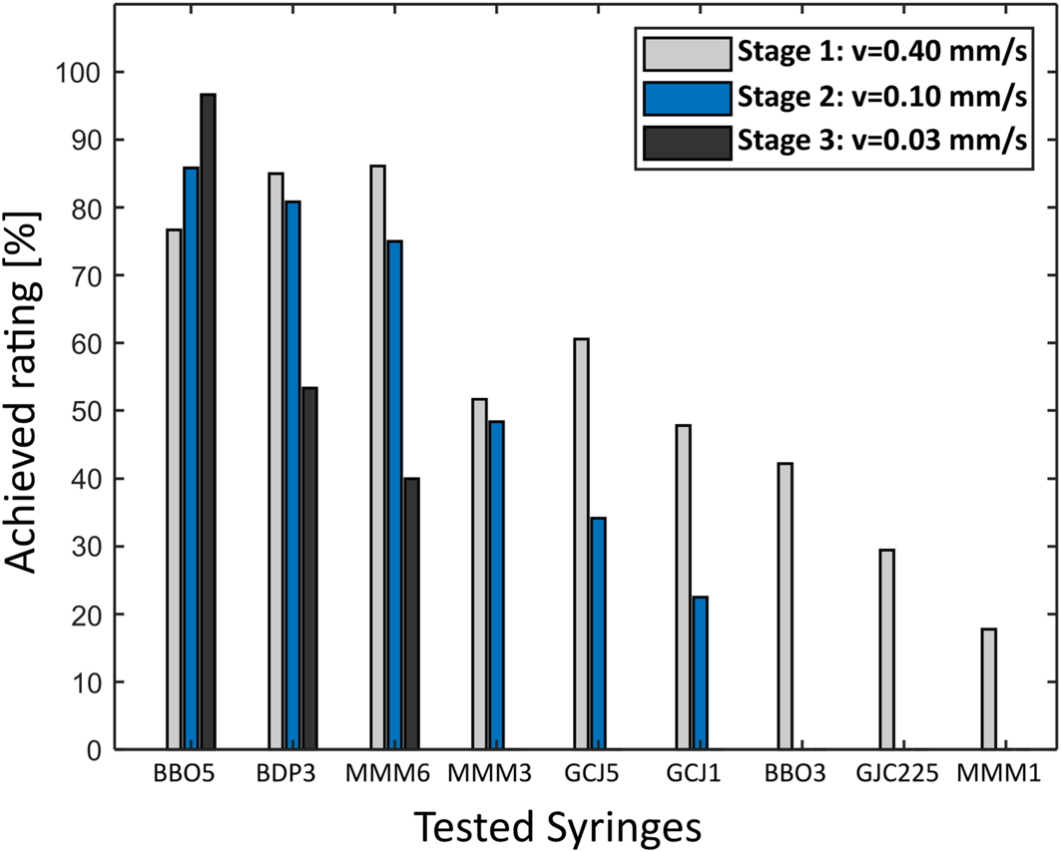
Results of the syringe selection experiments: syringe rating compared to best possible result for each stage

The rating results of the syringes are shown in Fig. 3. The syringe models BBO1 and BDP1 are not listed in the figure, since they did not start moving before the overpressure control of the infusion pump interrupted the hydraulic flow. Consequently, they were excluded from the study. Finally, the BBO5 was selected as best syringe because it showed a very consistent velocity across all stages with negligibly small deviation from the target velocity and low worst-case deviations. Especially at lower velocities in stage two and three, the actuation behavior was characterized by no measurable initial peaks, resulting in a direct increase towards the targeted velocity (see Fig. 5a). These properties were also represented by the calculated evaluation parameters, leading to a third best rating in the first stage and the best rating in the two following stages. In addition, after multiple trials, no damage to the syringe as well as no significant difference in actuation properties could be detected. All evaluation parameters of the selected BBO5 syringe are shown in Table 2, the results of all other syringes are listed in the supplementary material to this work (see Supplementary Table 1, 2, and 3, Additional File 1).

**Table 2:** Results of all stages for the best tested syringe (BBO5)

	Weighting factor	Evaluation stage		
		1 (0.40 mm/s)	2 (0.10 mm/s)	3 (0.03 mm/s)
Initial peak height [mm/s]	0.10	0.415	No peak	No peak
Initial peak width [s]	0.20	0.907	No peak	No peak
Difference between set and actual mean velocity [mm/s]	0.20	0.012	0.005	0.001
Worst-case velocity deviation [mm/s]	0.25	0.510	0.162	0.083
Highest standard deviation [mm/s]	0.25	0.048	0.017	0.014

The results of the manual actuation experiments are shown in Fig. 5c,d. The data reveal a substantially higher average speed compared to the hydraulic actuation, along with lower reproducibility of the velocity, including sudden velocity changes. Additionally, the trials show a large variability between the individual test subjects regarding velocity and consistency.

### Advancement of the Cochlea Hydrodrive design

#### Design changes to the Cochlea Hydrodrive system

**Fig. 4:**
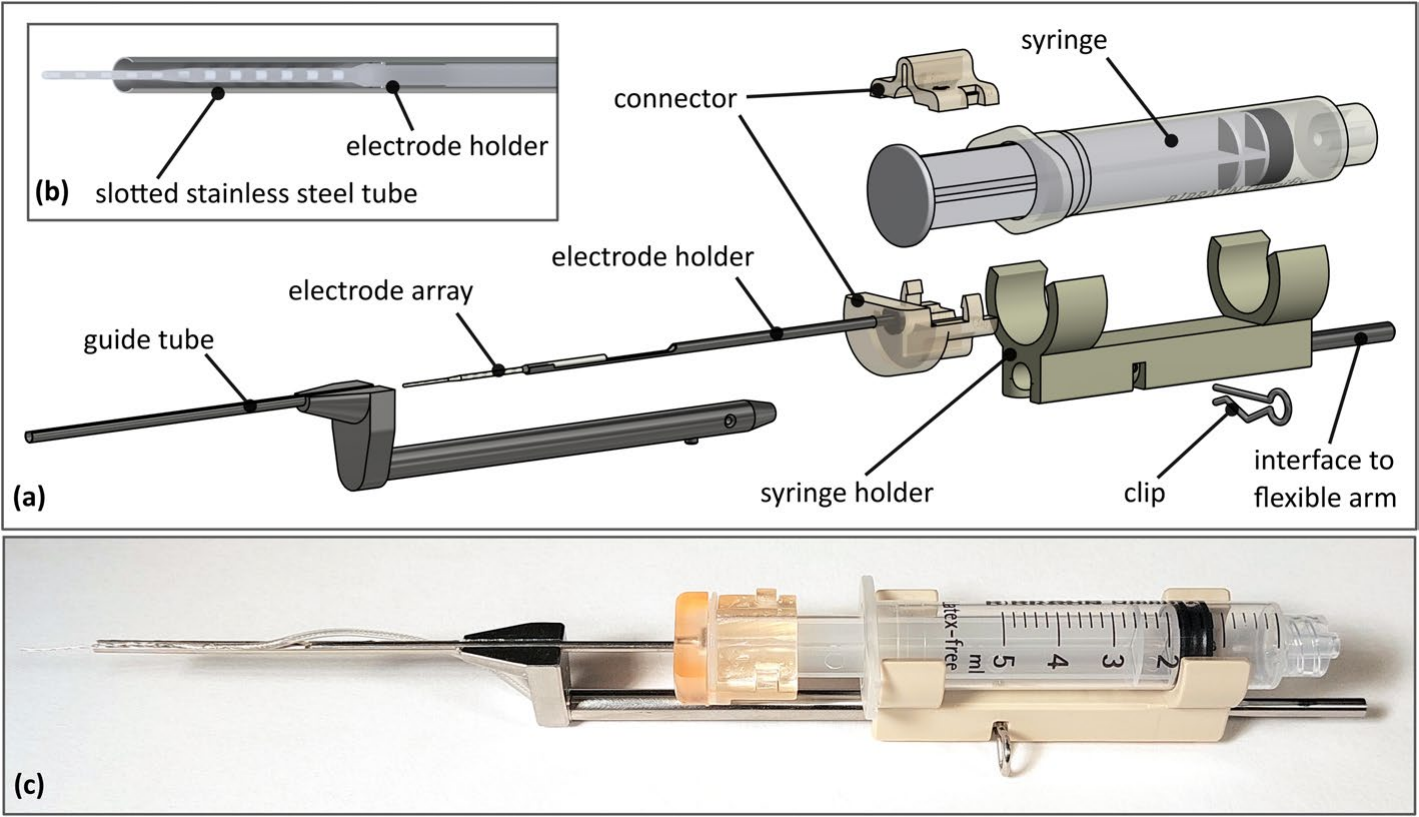
CHD design: **a** exploded isometric view of the CHD system with guide tube; **b** close-up rendering of the electrode holder within the guide tube; **c** manufactured and assembled final design of the CHD

Interestingly, the syringe model that proved to be best regarding the actuation properties is identical to the one that was used in the previous versions of the CHD design [27, 28]. Nevertheless, a number of design changes have been made in order to further improve the system (see Fig. 4a). The diameter of the electrode holder was reduced while the length was extended to reduce the size of the components located directly in the surgical site and thus to improve the field of vision of the surgeon. The syringe holder was slimmed down and adapted in a way that the syringe can be clamped into the holder from the side while ensuring a tight fixation of the syringe. The connector between the syringe and the electrode holder was also reduced in size for improved vision of the situs. Additionally, these two components are now fixed to each other via a clamping connection instead of a silicone rubber ring, further improving the ease of assembly. The most significant change, however, is the addition of an optional guide tube, which can be attached and secured to the syringe holder. The guide tube is a slotted stainless steel tube with an outer diameter of 2 mm, designed to allow the electrode holder to move forwards and backwards within it (see Fig. 4b). The slot allows the surgeon to remove the EA once the insertion is complete. The shape of the rear part of the guide tube component ensures that the syringe plunger can only advance to a point where the electrode holder does not exit the guide tube, effectively acting as an insertion depth limiter. Consequently, the guide tube could be positioned directly in front of the round window without the risk of the electrode holder crashing into the surgical site.

The components of the connector were 3D printed through stereolithography (Form 3B, Formlabs, Boston, USA) with a biocompatible, sterilizable resin (Surgical Guide, Formlabs, Boston, USA). The remaining parts were manufactured using surgical grade stainless steel and polyether ether ketone (PEEK), both of which are also autoclavable and biocompatible. The final manufactured design is shown in Fig. 4c.

#### Experimental evaluation—velocity profiles with guide tube

**Fig. 5:**
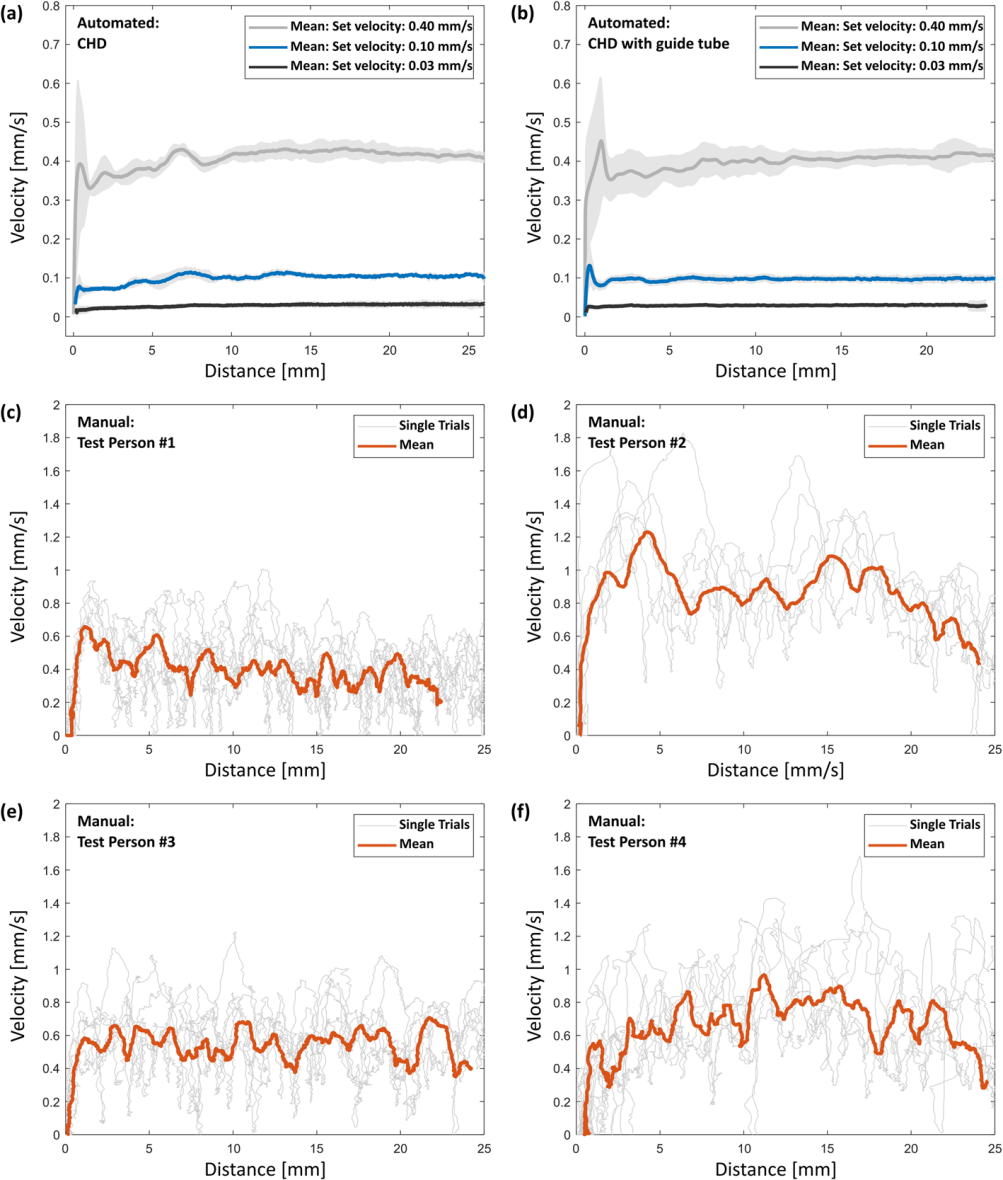
Overview over different actuation profiles. Note the different velocity ranges on the y-axis in **a** & **b** and **c**–**f**. **a** Mean velocity–distance profiles ± standard deviation from the best rated syringe (BBO5), i.e., the CHD without guide tube, for every tested velocity; **b** mean velocity–distance profiles ± standard deviation of the CHD system including the additional guide tube for every tested velocity; **c**–**f** actuation profiles of manual insertions performed by four different test persons using a surgical forceps

The guide tube was lubricated with small amounts of NaCl prior to each test. The actuation experiments showed a smooth and constant velocity of the CHD with guide tube (see Fig. 5b). Compared to the actuation profile without the added guide tube a slight initial peak was be observed. However, both the change of the syringe after every trial as well as the additional friction in the guide tube do not appear to have a negative influence on the smooth actuation behavior. Furthermore, the forward movement stopped abruptly when the maximum movement length was reached, showing that the insertion depth limiter is working as expected. Similar to the tests without the guide tube, no damage to the syringe due to the actuation could be detected.

#### Experimental evaluation—ex vivo insertion experiments

**Fig. 6:**
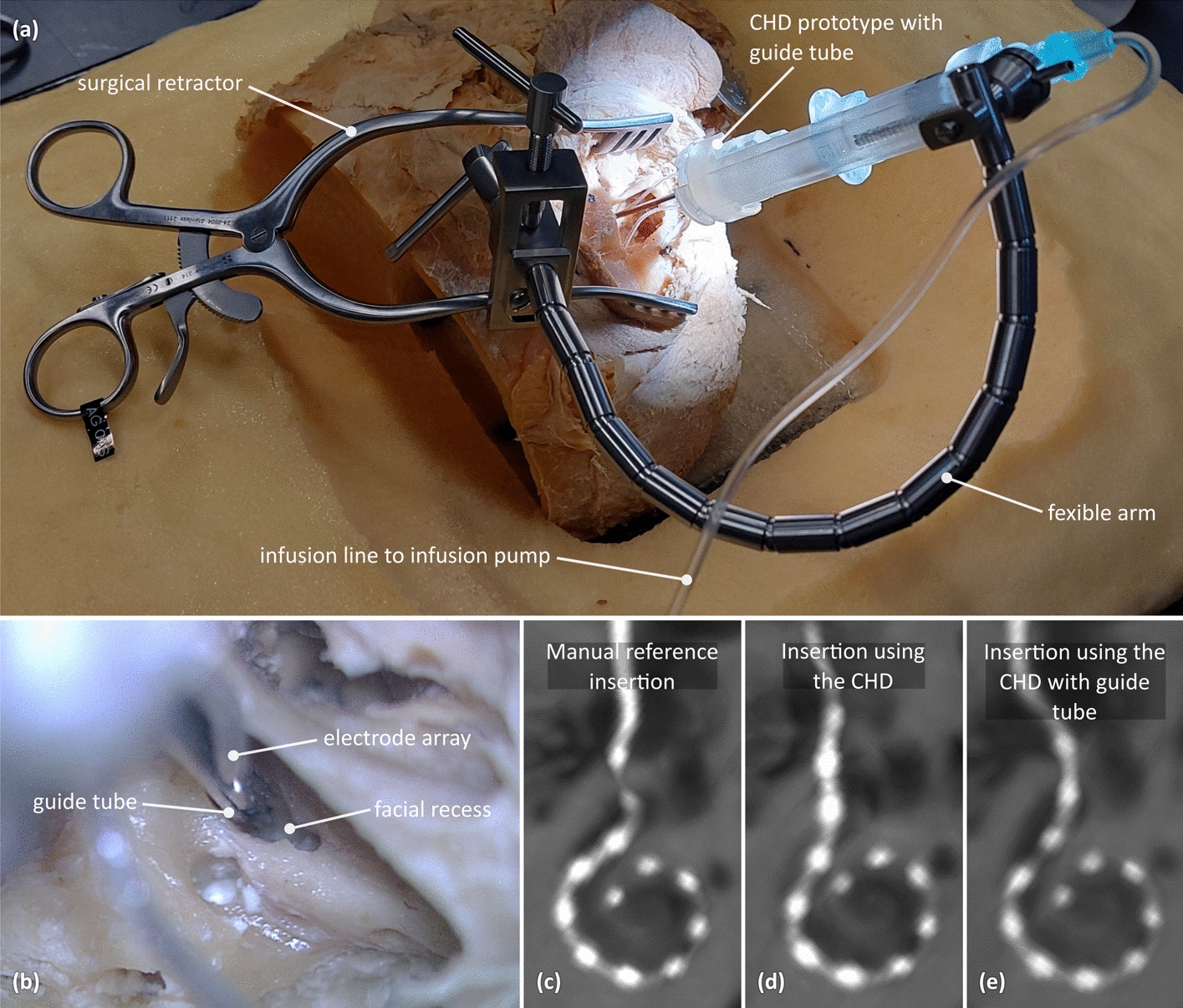
Insertion experiments. **a** CHD with guide tube fixed to the specimen head using a surgical retractor and a flexible arm. **b** Surgeon’s view through the microscope. Representative electrode positioning shown in the CBCT images after **c** manual reference insertion, insertion using **d** the CHD, and **e** the CHD with guide tube

All experimental ex vivo insertion trials were completed successfully. The CHD system could securely be fixed to the specimen head using the surgical retractor and the flexible arm (see Fig. 6a). Insertion of the EA into the scala tympani of the cochlea was achieved without requiring the surgeon to keep their hands on the CHD system, allowing necessary manual supporting steps, such as preventing EA buckling with a surgical claw, to be performed with both hands. Additionally, surgeons reported that using the CHD was intuitive and that their line of sight was not obstructed during the trials.

Both systems, the CHD with and without guide tube, proved to be highly effective for automated EA insertion. In trials using the additional guide tube, the CHD could be aligned with the tube being placed through the facial recess directly in front of the round window (see Fig. 6b). During the alignment, the guide tube shielded the EA, protecting it from surrounding bone and tissue. While EA buckling needed to be corrected using a second surgical tool during the automated insertion performed with the CHD without guide tube, no buckling event was observed when using the additional guide tube. The CBCT images of all trials indicated similar insertion depths between the automated procedures and the manual reference insertion (see Fig. 6c – d).

## Discussion

In this paper a motion tracking test setup was developed and evaluated which was used to analyze the hydraulic motion profiles of the CHD. This enabled the selection of the best syringe for the CHD using systematic evaluation parameters. Based on these findings, the CHD design was further adapted and advanced. The main advancement is the addition of a guide tube to surround the EA during the insertion. Ex vivo EA insertion experiments into cadaver specimens showed the overall feasibility of the automated procedure and the handling of the CHD, while also highlighting the benefits of the guide tube, particularly in terms of tool alignment and the prevention of EA buckling.

## Tracking test setup

The developed motion tracking system demonstrated high tracking accuracy for linear motion despite the use of a cost-effective camera. The observed outliers in the correlation and Bland–Altman plots of the velocity profiles can be attributed to a delay in tracking, primarily notable during phases of transition between forward movement and pauses. This delay may result from calculating the velocity plot as the average gradient within a 500 ms time window (15 frames at 30 fps). To enhance velocity tracking accuracy and reduce signal noise in the future, videos could be recorded at a higher frame rate. This would allow for decreasing the time period while keeping 15 frames for the derivation, thus decreasing the tracking delay. In addition, a higher-quality camera could be used, which would enable higher recording resolution and lower lens distortion rates.

The noise range of the motion tracking system with a confidence interval of ± 0.053 mm/s is within the range of the lowest tested velocity (0.03 mm/s). However, this confidence interval includes all velocities and pauses of the validation experiments, encompassing the transition phases with the outliers. When considering only the phases with constant velocity (steady speeds and pauses), the accuracy improves to a mean value of 0.009 mm/s with a confidence interval of ± 0.009 mm/s. This indicates that the measurement method is sufficiently accurate for constant velocities, even at the lowest tested speed. Furthermore, the method was employed primarily for comparative purposes between different syringes and systems. Even with a tracking delay, significant deviations and velocity peaks would still have been detected.

Moreover, our motion tracking accuracy results using a webcam (0.017 mm ± 0.017 mm) are superior to those reported in the literature using similar cost-effective cameras, ranging between a mean tracking accuracy of 0.13 mm [32] and 0.62 mm [33]. This could be attributed to the standardized conditions of our setup, including the fixed position of the webcam parallel and close to the marker pattern, the well-illuminated recording area and the predictable linear movement of the actuator in a single plane. However, the results still underscore the precision of our method in capturing detailed motion profiles and validate its sufficiency for characterizing the hydraulic velocity profiles.

## Syringe selection and hydraulic actuation

The chosen weighting factors (WF) of the evaluation parameters were determined according to their relative importance to the performance of the total system and the safety of the patient. The parameters given the highest impact were the standard deviation and the worst-case value, each assigned a WF of 0.25. This decision was made since we wanted the system to perform a very steady movement without velocity peaks which could potentially introduce a high impulse to the cochlea and therefore could damage inner structures [34]. The initial peak height was assigned the lowest WF (0.1). At the beginning of the insertion the EA is either outside the scala tympani or has just entered the basal turn having minimal or no contact with surrounding structures. A significant impact caused by quasistatic pressure is also not expected with a sufficiently large round window opening [20]. Therefore, a high but short peak is not expected to exert a significant influence on intrascalar structures. However, a sustained high peak over a longer period would be more critical. In such cases, the initial peak width parameter (WF: 0.2) would drastically decrease the performance rating value of the syringe. The chosen evaluation parameters and WF for objective syringe selection proved to be effective in identifying the best actuation properties as syringes with obviously poor characteristics received correspondingly inferior ratings and were quickly eliminated.

Some syringe models showed a poorer performance at slower velocities, primarily due to stick–slip events, which mainly occur when static friction is higher than kinetic friction [35]. This phenomenon is more likely to occur at lower speeds, where the pressure force in the syringe at the set velocity might not be sufficient to overcome static friction, resulting in movement only when sufficient pressure builds to surpass static friction before slowing down upon transitioning to kinetic friction. This highlights the benefit of testing the syringes at different velocities to assess the magnitude of stick–slip effects. Using an alternative hydraulic fluid, such as hydraulic oil, could potentially improve actuation behavior and reduce stick–slip effects. However, due to the more complex requirements for sterility and biocompatibility, and considering the satisfactory actuation results achieved, we did not to explore this option further.

Compared to our previous work where the average velocity was estimated based on the time it took to travel a fixed distance [27], we were now able to evaluate the complete velocity–distance profiles of the CHD system over its whole movement range. These results reveal a highly accurate and smooth motion for all tested velocities, especially when comparing it to manual actuation (see Fig. 5c to f), where repeatability and uniformity are subject to strong variations. In addition, the CHD provides reliable linear actuation at ultra-slow velocities such as 0.03 mm/s, which other automated insertion devices on the market are not yet capable of performing [36]. This velocity is also significantly lower than the average velocity in slow manual insertions performed by surgeons reported in literature, ranging between 0.87 mm/s and 0.19 mm/s [37, 38]. The smooth profile and execution of a predefined velocity shows the advantages compared to manual insertions and conclusively demonstrates the feasibility and safety of this approach using hydraulic actuation as part of an automated EA insertion device.

## CHD design and experimental results

To further enhance standardization, precision and safety of the EA insertion process, our study explored the use of a guide tube with the CHD. This general idea is not new, as guide tubes have been used in automated insertion test setups in order to provide a higher degree of standardization and high repeatability of insertion behavior of the EA [19, 39–41]. By preventing the electrode from buckling or kinking before entering the scala tympani, the standard deviation in force measurements remains small, allowing for high reproducibility of the results [41]. In manual treatment, surgeons aim to limit basal electrode buckling by moving their hand or manipulating the array with additional surgical tools, e.g., surgical claws and needles [42], as electrode buckling exposes uncontrolled elastic forces onto the inner structures of the scala tympani, which are believed to drive insertion forces [20].

Several studies also introduced guide tubes for use in minimally invasive CI surgery. These devices have been employed either as part of a manual tool to insert the EA [43, 44] or as an additional tube which is inserted into the drill canal prior to insertion [45–47] to shield the EA during its transition through the drill canal and guide it through the middle ear right to the cochlear opening. The latter is already considered a part of the clinical workflow for minimally invasive CI surgery [48]. Despite these benefits, current automated insertion devices available on the market do not feature this type of add-on, which facilitates EA guidance through the facial recess right in front of the round window in the standard surgical approach [36].

The introduced guide tube addresses this gap by offering an optional add-on for the CHD system, in order to enhance standardization of the EA insertion process. With a diameter of 2 mm the guide tube fits through the facial recess in the majority of patients, as the mean width of the facial recess at the level of the round window is reported in literature as 2.65 mm ± 0.41 mm [49]. In our experimental trials, no further adaptations or extensions to the posterior tympanotomy and round window were necessary beyond the standard surgical access. In case that the guide tube does not fit through the facial recess up to the cochlea opening due to a narrow individual anatomy, it can still be placed right in front of the posterior tympanotomy. Alternatively, the CHD can be used without the guide tube, as this feature is designed to be dismountable. The insertion would still benefit from steady and smooth forward movement.

The insertions into the cadaver specimens were evaluated successful in all cases. The slightly decreased insertion depth compared to a full insertion using the CHD was in line with the manual reference insertion and can be attributed to a difference in tissue behavior and friction conditions of the preserved cadaver specimens. The repeated insertions with the EAs likely did not influence the insertion results. While an initial softening of the EA due to conditioning may be observed, this effect remains stable over time, and repeated testing under these conditions typically does not affect the wear of the EA [41]. Furthermore, no damage to the EA was observed upon visual inspection with the microscope after testing. In general, these insertion trials confirmed the benefits of the guide tube especially in two aspects. Firstly, the guide tube simplifies the alignment process, due to its placement through the facial recess directly in front of the round window. As the guide tube represents and therefore “visualizes” the insertion trajectory, it is easier to correctly align the tool along the desired trajectory and therefore reduces the need to correct the insertion trajectory during the insertion process once the forward movement is started. This is beneficial, as reaching the optimal insertion trajectory in robot assisted EA insertion is reported challenging in literature, especially when using ultra-soft EAs [50]. By orienting the slit of the guide tube anterior to the cochlear opening and bone groove in the facial recess, decoupling of the electrode and fixing it to the groove was positively evaluated during all experiments. The second major advantage of the guide tube shown in the trials is the effectiveness in preventing EA buckling, which in turn contributes to the benefits described above. This further enhances the standardization of the process, since the surgeons did not need to intervene and perform manual correction steps, even in the presence of the specimen's challenging tissue characteristics. Furthermore, the EA did not buckle out of the slot in the guide tube during the four insertions performed into the cadaver specimen. This is likely caused by the surface tension of NaCl droplets from initial flushing of the tube, which helps to keep the flexible EA aligned with the tube wall.

Besides these promising results it should be noted that only a limited number of trials were conducted on cadaver head specimens and further tests are needed to evaluate the generalizability of these findings. However, while our study did not show enhancement of the insertion depth or further smoothing of the motion profile using the guide tube compared to the CHD without guide tube, its advantages in improving the alignment process and preventing EA buckling make a compelling case for its use.

## Conclusion

In summary, this study provided further insights into the hydraulic actuation properties of the *Cochlea Hydrodrive* (CHD) through the use of a newly developed motion tracking test setup. The data obtained demonstrated highly accurate, smooth, and reliable motion profiles for velocities as low as 0.03 mm/s. Additionally, the CHD system was enhanced with a guide tube and subsequently evaluated in ex vivo insertion experiments. The guide tube proved beneficial for the insertion procedure, improving tool alignment and reducing electrode buckling. This work represents a significant step towards the clinical translation of the tool, demonstrating its safety and usability for robotic electrode insertion in cochlear implant surgery while enabling a higher degree of standardization.

## Supplementary Information

Supplementary Material 1. Table 1 Hydraulic actuation results for all tested syringes for stage one. Syringes placed at ranks one to six were transferred to the next stage. Table 2 Hydraulic actuation results for all tested syringes for stage two. Syringes placed at ranks one to three were transferred to the next stage. Table 3 Hydraulic actuation results for all tested syringes for stage three

## Data Availability

The data that support the findings of this study are available in the supplementary material and from the corresponding author upon reasonable request.

## Declarations

### Generative AI and AI-assisted technologies in the writing process

Parts of the manuscript were improved by using artificial intelligence (ChatGPT 3.5) for spell checking and improving readability of single sentences. After carefully reviewing, the authors confirm that the algorithm only corrects grammar errors and typos while keeping the original meaning of the statements. The authors take full responsibility for the content of the publication.
