# Supplementary material for: Preclinical evaluation of a hydraulic actuation system with guide tube for robotic cochlear implant electrode insertion
Source: Biomed Eng Online. 2025 Feb 14;24:19. doi: 10.1186/s12938-025-01338-z (PMC11829445; doi:10.1186/s12938-025-01338-z)
Supplement: Supplementary file 1 — Supplementary Material 1. Table 1 Hydraulic actuation results for all tested syringes for stage one. Syringes placed at ranks one to six were transferred to the next stage. Table 2 Hydraulic actuation results for all tested syringes for stage two. Syringes placed at ranks one to three were transferred to the next stage. Table 3 Hydraulic actuation results for all tested syringes for stage three [file 12938_2025_1338_MOESM1_ESM.docx]

# Additional File 1

This supplementary material belongs to the manuscript: *Preclinical Evaluation of a Hydraulic Actuation System with Guide Tube for Robotic Cochlear Implant Electrode Insertion.*

Jakob Cramer^1,2^, Rolf Salcher^1^, Max Fröhlich^1,3^, Georg Böttcher-Rebmann^1,2^, Eralp Artukarslan^1^, Thomas Lenarz^1,2^ and Thomas S. Rau^1,2^

# Results – Hydraulic Actuation

In the following Supplementary Tables I to III, the results of the hydraulic actuation evaluation parameters for all tested syringes across all three stages are presented. The total rank indicates the performance of each syringe after multiplying the weighting factors to the parameters, with 1^st^ place representing the best-performing syringe in each stage.

Table I: Hydraulic actuation results for all tested syringes for stage one (v = 0.4 mm/s). Syringes placed at ranks one to six were transferred to the next stage.

| **Syringe Type** | **Initial peak height [mm/s]** | **Initial peak width [s]** | **Difference between set and actual mean velocity [mm/s]** | **Worst case velocity deviation [mm/s]** | **Highest standard deviation [mm/s]** | **Total**  **Rank** |
| --- | --- | --- | --- | --- | --- | --- |
| MMM6 | 0.707 | 2.134 | 0.001 | 0.463 | 0.032 | **1** |
| BDP3 | 0.445 | 3.139 | 0.007 | 0.470 | 0.029 | **2** |
| BBO5 | 0.415 | 0.907 | 0.012 | 0.510 | 0.048 | **3** |
| GCJ5 | 0.891 | 5.017 | 0.016 | 0.567 | 0.043 | **4** |
| MMM3 | 0.573 | 1.693 | 0.017 | 0.649 | 0.191 | **5** |
| GCJ1 | 1.685 | 16.059 | 0.010 | 0.612 | 0.048 | **6** |
| BBO3 | 0.608 | 1.413 | 0.024 | 2.229 | 0.509 | **7** |
| GCJ225 | 1.624 | 11.816 | 0.039 | 0.833 | 0.049 | **8** |
| MMM1 | 2.036 | 9.135 | 0.036 | 3.086 | 0.660 | **9** |
| BBO1 | Excluded: No motion possible | | | | | **--** |
| BDP1 | Excluded: No motion possible | | | | | **--** |

Table II: Hydraulic actuation results for all tested syringes for stage two (v = 0.1 mm/s). Syringes placed at ranks one to three were transferred to the next stage.

| **Syringe Type** | **Initial peak height [mm/s]** | **Initial peak width [s]** | **Difference between set and actual mean velocity [mm/s]** | **Worst case velocity deviation [mm/s]** | **Highest standard deviation [mm/s]** | **Total**  **Rank** |
| --- | --- | --- | --- | --- | --- | --- |
| BBO5 | No peak | No peak | 0.005 | 0.162 | 0.017 | **1** |
| BDP3 | 0.158 | 1.496 | 0.001 | 0.160 | 0.018 | **2** |
| MMM6 | 0.633 | 1.239 | 0.000 | 0.606 | 0.174 | **3** |
| MMM3 | 0.600 | 1.938 | 0.009 | 0.692 | 0.189 | **4** |
| GCJ5 | 1.328 | 4.082 | 0.001 | 2.259 | 0.332 | **5** |
| GCJ1 | 1.131 | 11.494 | 0.028 | 2.204 | 0.779 | **6** |

Table III: Hydraulic actuation results for all tested syringes for stage three (v = 0.03 mm/s).

| **Syringe Type** | **Initial peak height [mm/s]** | **Initial peak width [s]** | **Difference between set and actual mean velocity [mm/s]** | **Worst case velocity deviation [mm/s]** | **Highest standard deviation [mm/s]** | **Total**  **Rank** |
| --- | --- | --- | --- | --- | --- | --- |
| BBO5 | No peak | No peak | 0.001 | 0.083 | 0.014 | **1** |
| BDP3 | 0.045 | 2.196 | 0.001 | 0.267 | 0.037 | **2** |
| MMM6 | 0.431 | 0.801 | 0.001 | 0.437 | 0.089 | **3** |
